# Preparation of fluoropolymer materials with different porous morphologies by an emulsion template method using supercritical carbon dioxide as a medium†

**DOI:** 10.1039/c9ra00777f

**Published:** 2019-04-11

**Authors:** Jian Chen, Umair Azhar, Yongkang Wang, Jihong Liang, Bing Geng

**Affiliations:** Shandong Provincial Key Laboratory of Fluorine Chemistry and Chemical Materials China; Institute of Fluorescent Probes for Biological Imaging, University of Jinan Shandong China chm_gengb@ujn.edu.cn; School of Chemistry and Chemical Engineering, University of Jinan Jinan 250022 China

## Abstract

The choice of a suitable surfactant is key to the formation of a stable water-in-CO_2_ (W/C) or CO_2_-in-water (C/W) emulsion. It is even more critical in stabilization of the emulsion containing carbon dioxide (CO_2_). In this study, the successful preparation of W/C emulsion was achieved by using the amphiphilic block polymer poly(ethylene glycol) methyl ether-*b*-poly(trifluoroethyl methacrylate) (mPEG_45_-*b*-(TFEMA)_*n*_) as a surfactant, in which CO_2_ was used as a solvent for the fluoromonomer, trifluoroethyl methacrylate (TFEMA). In the case of the W/C emulsion, CO_2_ and TFEMA were used as the continuous phase and water as the internal phase of the emulsion system. It has been found that in the length of the block polymer mPEG_45_-*b*-(TFEMA)_*n*_, the fluorine-containing chain end has a significant effect on the morphology of the polymer and the type of emulsion formed. The morphology of the polymer was observed by scanning electron microscopy which confirmed the type of emulsion formed. With the fluorine-containing end segment, the morphology of the polymer changes from a small hollow sphere in a large hollow sphere to a hollow spherical to a porous structure. Correspondingly, it could be concluded that the type of emulsion could go through the process from water-in-CO_2_-in-water-in-CO_2_ (W/C/W/C) emulsion to water-in-CO_2_-in-water (W/C/W) emulsion to water-in-CO_2_ (W/C) emulsion. Also, suitable co-surfactants were identified in this study. Investigations were also attempted to check the effect of the amount of surfactant, cross-linker and water/CO_2_ ratio on the type of emulsion formed as well as the morphology of the resultant polymer.

## Abstract 2

Effect of the length of the block polymer mPEG_45_-*b*-(TFEMA)_*n*_, fluorine-containing chain end on final polymer morphology.

## Graphical abstract

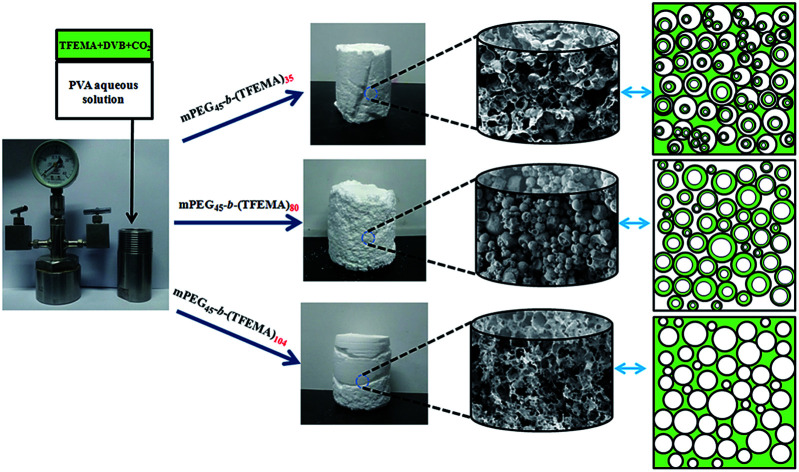

## 1. Introduction

Porous polymers are widely used in tissue engineering,^1,2^ gas storage and separation,^3–5^ catalyst supports,^6–9^ heavy metal ion collectors^10^ and oil–water separation,^11^ owing to their large pore volume, good porosity and interconnected morphology. The emulsion template method can produce well-defined porous polymers and inorganic materials. It effectively makes a porous structure by making an oil-in-water (O/W) and water-in-oil (W/O) emulsion, which is simple and easy to prepare.

Supercritical carbon dioxide (sc-CO_2_) is a non-toxic, non-flammable, inexpensive natural solvent^12^ and possesses moderate critical temperature and pressure (*T*_c_ = 31.1 °C, *P*_c_ = 7.38 MPa).^13^ It can be used as a substitute for conventional organic solvents for the preparation of water-in-carbon dioxide (W/C) emulsions or carbon dioxide-in-water (C/W) emulsions. Moreover, CO_2_ has a lower critical pressure and temperature, and when used as a solvent, it can be removed by changing the temperature and pressure, which makes the post-treatment simple.^14^ But, CO_2_ is usually a very poor solvent with a low van der Waals forces and dielectric constant.^15^ Thus, it has a very low solubility for hydrophilic molecules, high molecular weight substances, and metal ions, which limits its use on wide range of applications. However, fluoromonomers have good compatibility with CO_2_, and fluorination is also considered to be a key feature for generating CO_2_-philic surfactants.^16,17^ In general, CO_2_ can be used as a good solvent for fluoromonomers. Also, the fluorine atom has strong electronegativity, low polarizability, weak van der Waals forces and possess high C–F bond energy (485.3 KJ mol^−1^) has high energy. All of these characteristics of fluorine atom make fluoropolymer to have excellent chemical properties, such as high weather resistance, high heat resistance, and high stability. Fluoromonomers are also expensive and large volume polymeric materials can be prepared by the emulsion template method using solvent addition, which ultimately decreases costs.

Sc-CO_2_ is used in a large number of applications in emulsion polymerization, which mainly acts as a template for the internal phase to prepare C/W emulsion, or acts as a continuous phase to prepare W/C emulsion. The application of C/W emulsions in the preparation of porous polymeric materials is more extensive. Boyere *et al.*^18^ synthesized a new non-ionic surfactant, fluorinated glycosurfactant, which acts to emulsify CO_2_/H_2_O system and formed a stable C/W high internal phase emulsion (HIPE). A poly(acrylamide) (PAM) material having a cross-linked porous structure was obtained by polymerization. Partap *et al.*^19^ produced emulsion templated alginate hydrogels by using a surfactant ammonium perfluoropolyether (PFPE-NH_4_), where CO_2_ not only served as a templating agent but also as a reagent by increasing the acidity of the aqueous phase to initiate the gelation of alginate. Butler *et al.*^20^ used perfluoropolyether (PFPE) as a surfactant and poly(vinyl alcohol) (PVA) as a co-surfactant to stabilize C/W emulsions. Without adding of PVA, it became difficult to stabilize C/W emulsion containing monomer. However, a stable C/W emulsion can be prepared in the presence of PVA. Luo *et al.*^21,22^ successfully prepared stable C/W emulsions only by using PVA as a stabilizer without addition of any conventional surfactants. It has been proved that different types of PVA and its concentrations can affect the stability of the emulsion. Thus, it has to be noted that fluorosurfactants are widely used in the preparation of C/W emulsions, and it can be successfully used to prepare porous materials by the emulsion template method. It is also evident that PVA is widely used as a co-stabilizer in the emulsion polymerization.

W/C emulsions have also been widely reported as compared to C/W emulsions. From the first preparation of W/C reverse microemulsion by Harrison in 1994,^23^ W/C emulsions have been extensively used in a wide range of applications, as they easily be utilized in the dispersion of medicines^24^ and metal salts in supercritical carbon dioxide,^25^ organic synthesis^26^ and preparation of nanoparticles.^27^

Controlled/living radical polymerization (CLRP) has been widely used as it is now possible to synthesize a wide variety of previously inaccessible macromolecules under relatively mild conditions. CLRP includes nitroxide-mediated polymerization, atom transfer radical polymerization (ATRP) and reversible addition–fragmentation polymerization (RAFT).^28^ This technology has been used in preparation of many semi-fluorinated polymers. Discekici *et al.*^29^ synthesized a semifluoride with a low molar mass dispersity and excellent chain-end fidelity by a versatile light-mediated ATRP protocol. Anastasaki *et al.*^30^ reported a one-pot synthesis of multi-block copolymers using hydrophobic, hydrophilic and fluorinated monomers by a light-mediated ATRP protocol, and their route generated high monomer conversion, low dispersities and fully retained end groups to allow for multiple chain extensions. RAFT polymerization has distinct advantages compared with ATRP. It can be used to prepare polymers or copolymers with narrow molecular weight distribution. Molecular weight of the final product can be anticipated from the ratio of monomer consumed to chain transfer agent (CTA). In addition there is no undesired metal species introduced during RAFT polymerization process.^31^ Truong *et al.*^32,33^ synthesized thermo-responsive copolymer P(DEGMA-*co*-HPMA), then they described its application in the RAFT-mediated emulsion polymerization of styrene in order to produce nanoparticles with tuneable morphology.

The porous polymeric materials with superb oleophilic and hydrophobic nature exhibit high oil/water separation efficiency. These fluoropolymers with controllable morphologies are vital in many applications of environmental concerns such as chemical resistive oil adsorbents, dye adsorbents, among others. The foremost requirement of the good oil adsorbent is that its surface should be composed of low surface energy materials. Fluoropolymers such as poly(vinylidene fluoride), poly(hexafluorobutyl acrylate), and poly(tetrafluoroethylene) provide efficient hydrophobic and oleophilic surfaces owing to the very low surface energy of –CF_2_– groups. In the preparation process of P(TFEMA–DVB) foam *via* HIPE templating, fluorine atoms which are hydrophobic in nature dispersed on the pores surfaces which make them good choice to use in oil adsorption without penetration of water inside the pores.^11^ Recently, stable W/O HIPE fluorinated emulsions were successfully prepared by using a commercial surfactant Hypermer B246 (ref. 11) and a cationic diblock copolymer (PDMAEMA-*b*-PHFBA),^34^ to obtain high performance fluoropolymer foams. However, Hypermer B246 is a diblock polymer, and it is difficult to obtain a precise effect of the length of CO_2_-philic chain segment on the morphology of the polymer. In this investigation, a diblock copolymer (mPEG_45_-*b*-(TFEMA)_*n*_) was synthesized to prepare a stable W/C emulsion. The length of the block polymer mPEG_45_-*b*-(TFEMA)_*n*_ CO_2_-philic chain segment (fluorine-containing segment) is controlled by RAFT polymerization. By using this type of fluorosurfactant diblock copolymer, a series of fluorinated materials were prepared with (2,2,2-trifluoroethyl methacrylate) (TFEMA) as a primary monomer, and divinylbenzene (DVB) as a crosslinker. Polymers with different morphologies were obtained by changing the size of “*n*” in the block polymer, where mPEG_45_-*b*-(TFEMA)_*n*_ acted as surfactants, to obtain different types of emulsion. By this the morphology of the polymer, poly(TFEMA–DVB) could be easily tuned. Through a series of experiments, the morphology of the polymer with a small hollow sphere in the large hollow sphere, a hollow spherical and porous structure was obtained, and the differences in the adsorption of different solvents were explored.

## 2. Experimental

### 2.1 Materials

The monomer, 2,2,2-trifluoroethyl methacrylate (TFEMA, hydrophobic monomer), was purchased from Weihai Newera Chemical Co, Ltd. The crosslinker, divinylbenzene (DVB, technical grade, 80%) and azobisisobutyronitrile (AIBN) were purchased from Sigma-Aldrich (USA). Hypermer 70 (A70) was purchased from Croda. Sorbitan stearate (Span 60) was obtained from Sigma-Aldrich (USA). Polyoxyethylene sorbitan monooleate (Tween-80), cationic surfactant hexadecyltrimethylammonium bromide (CTAB), anionic surfactant sodium dodecyl sulfate (SDS) were provided by Sinopharm Chemical Reagent Co., Ltd (Shanghai, China). PVA (99% hydrolyzed, 74 800 g mol^−1^ (1799) and 114 400 g mol^−1^ (2699)) and PVA (88% hydrolyzed, 74 800 g mol^−1^ (1788) and 105 600 g mol^−1^ (2488)) were purchased from Sigma-Aldrich. *N*,*N*′-Dicyclohexylcarbodiimide (DCC, Aladdin), 4-dimethylaminopyridine (DMAP, Aladdin), dichloromethane (DCM, 99.9%) and 1,4-dioxane (99.5%) were supplied by Tianjin Fuyu Fine Chemical Company. *S*-1-Dodecyl-*S*′-(α,α′-dimethyl-α′′-acetic acid)trithiocarbonate (DDMAT) as the RAFT agent was synthesized based on the previous report.^35^

### 2.2 Synthesis of poly(ethylene glycol) methyl ether macro-chain transfer agent (mPEG_45_-DDMAT)

**Scheme 1:**
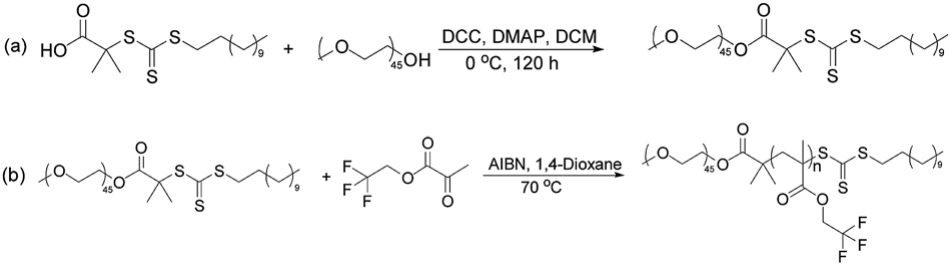
Preparation of block polymer mPEG_45_-*b*-(TFEMA)_*n*_.

The synthesis of poly(ethylene glycol) methyl ether macro-chain transfer agent (macro-CTA) was conducted as follows. At first, to a 200 ml round bottom flask poly(ethylene glycol) methyl ether (mPEG_45_-OH, 10 g, 5 × 10^−3^ mol), *S*-1-dodecyl-*S*′-(α,α′-dimethyl-α′′-acetic acid) trithiocarbonate (DDMAT; 4.5 g, 0.0124 mol), 4-dimethylaminopyridine (DMAP; 0.15 g; 1.23 × 10^−3^ mol), dichloromethane (DCM; 18 ml) were charged. A mixed solution of 2.5 g DCC and 15 ml DCM was added to the constant pressure dropping funnel. Then, the solutions were added for about 20 min by titration under ice water bath to make the reaction uniform. The flask was then placed in a magnetic stirrer and stirring was continued at 0 °C for 120 h. The reaction solution was then concentrated, and slowly dropped into a 1000 ml beaker containing 800 ml of anhydrous diethyl ether to induce the precipitation (yield 72.1%). The followed process is shown in Scheme 1(a).

### 2.3 Synthesis of poly(ethylene glycol) methyl ether-*b*-poly(trifluoroethyl methacrylate) (mPEG_45_-*b*-(TFEMA)_*n*_)

The RAFT macro agent mPEG_45_-DDMAT (0.3518 g, 1.49 × 10^−4^ mol), 2,2,2-trifluoroethyl methacrylate (TFEMA; 5 g; 0.0298 mol) and 2,2′-azobisisobutyronitrile (AIBN; 0.0086 g, 5.24 × 10^−5^ mol, and the molar ratio of macro-CTA/AIBN = 3) were dissolved in 1,4-dioxane (20 g). The reaction mixture was sealed in a 50 ml round bottom flask and kept in a magnetic stirrer and purged with nitrogen gas to completely remove oxygen from the vessel. The deoxygenated solution was then placed in a preheated water bath at 70 °C for 24 h with agitation. The utilized process is shown in Scheme 1(b).

### 2.4 Preparation of stable emulsions and fluoropolymer materials

**Fig. 1:**
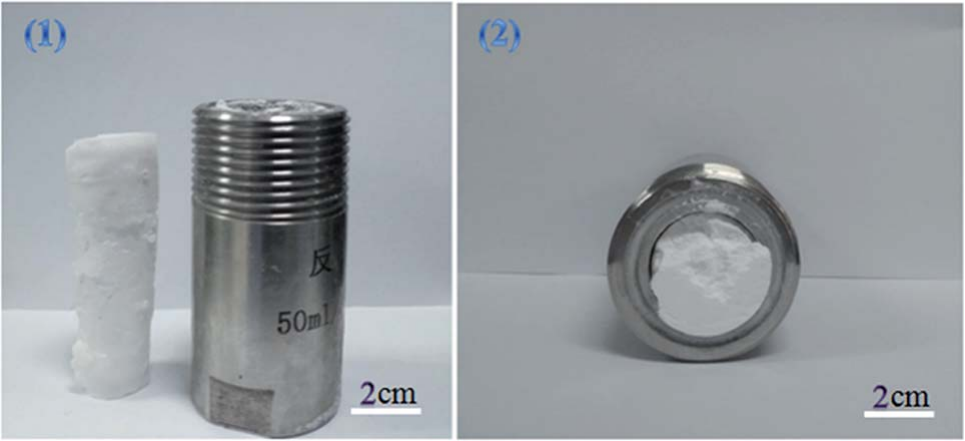
Photographs of a polymer poly(TFEMA–DVB) formed by W/C emulsion: (1) (right) high-pressure reactor used in this experiment and (left) sample (2) the sample inside the autoclave.

TFEMA (3.5 g), DVB (0.39 g), 2,2′-azobisisobutyronitrile (AIBN) (0.0039) g, and 0.39 g of block polymer mPEG_45_-*b*-(TFEMA)_*n*_ were added to the serum bottle, and ultrasonically dispersed to form an uniform oil phase. Polyvinyl alcohol (PVA) was dissolved in deionized water at 80 °C to prepare an aqueous solution of polyvinyl alcohol (1.5% by mass), and 38 ml of polyvinyl alcohol solution was taken and added to a 50 ml autoclave. The prepared oil phase was added to an autoclave containing PVA solution, the autoclave was sealed and evacuated, charged with CO_2_ (10 g), pre-stirred for 30 min under magnetic stirring, slowly heated to 70 °C with continuous stirring for 40 min, and then in the absence of magnetic stirring reaction was carried out at 70 °C for 6 h. CO_2_ was discharged, and the complete columnar fluoropolymer material was taken out (Fig. 1). Other compositions of the emulsions for the preparation of poly(TFEMA–DVB) are shown in Table 1.

**Table 1:** Composition for each group of experiments

Sample^a^	TFEMA/DVB mass ratio	Surfactant (wt%)	CO_2_ (g)	PVA aqueous (1.5% mass fraction) (ml)
B1	0.9	10^b^	10	38^g^
B2	0.9	10^c^	10	38^g^
B3	0.9	10^d^	10	38^g^
B4	0.9	10^e^	10	38^g^
B5	0.9	10^f^	10	38^g^
C1	0.9	10^e^	10	38^h^
C2	0.9	10^e^	10	38^g^
C3	0.9	10^e^	10	38^i^
C4	0.9	10^e^	10	38^j^
D1	0.9	3^e^	10	38^g^
D2	0.9	5^e^	10	38^g^
D3	0.9	15^e^	10	38^g^
D4	0.9	20^e^	10	38^g^
E1	0.8	10^e^	10	38^g^
E2	0.7	10^e^	10	38^g^
E3	0.5	10^e^	10	38^g^
F1	0.9	10^e^	10	15^g^
F2	0.9	10^e^	10	25^g^
F3	0.9	10^e^	10	30^g^
G1	0.9	10^k^	10	38^g^
G2	0.9	10^l^	10	38^g^

a For all the samples, AIBN was 2 wt% with respect to the total oil phase (TFEMA and DVB).

b Using block polymer mPEG_45_-*b*-(TFEMA)_35_ as a surfactant.

c Using block polymer mPEG_45_-*b*-(TFEMA)_45_ as a surfactant.

d Using block polymer mPEG_45_-*b*-(TFEMA)_80_ as a surfactant.

e Using block polymer mPEG_45_-*b*-(TFEMA)_104_ as a surfactant.

f Using block polymer mPEG_45_-*b*-(TFEMA)_150_ as a surfactant.

g Using 2488 PVA as a co-surfactant.

h Using 1788 PVA as a co-surfactant.

i Using 1799 PVA as a co-surfactant.

j Using 2699 PVA as a co-surfactant.

k Using block polymer mPEG_45_-*b*-(TFEMA)_*n*_ prepared by macro-CTA/AIBN = 1.

l Using block polymer mPEG_45_-*b*-(TFEMA)_*n*_ prepared by macro-CTA/AIBN = 2.

## 3. Characterization

Approximately 0.4 cm^3^ pieces of monoliths were adhered to the conductive tape using a silver paste before subjecting to scanning electron microscopy (SEM) (S-2500, Hitachi Seiki Ltd., Japan) to check the morphology of the polymer. A sample with good conductivity was obtained by spraying gold at a current of 75 mA for 120 s. The hole size was measured using a Nano Measurer 1.2 software. ^1^H NMR spectra were recorded in acetone using Bruker Advance III 400 MHz nuclear magnetic resonance spectrometer at room temperature and the molecular weight of the block polymer was finally determined. The number average molecular weight (*M*_n_) and polydispersity index (PDI) of the block polymer (mPEG_45_-*b*-(TFEMA)_*n*_) were analyzed by gel permeation chromatography (GPC), where THF as a mobile phase with a flow rate of 1 ml min^−1^ was used. The system was equipped with a Waters Model 1525 HPLC pump and a Waters Model 2414 refractive index detector. Fourier-transform infrared spectroscopy (FTIR) of the polymer poly(TFEMA–DVB) was conducted on a FTIR Nicolet iS10 spectrophotometer from 4000 to 500 cm^−1^. The specific surface area of the porous polymer was measured by using the nitrogen adsorption isotherm and by employing the Brunauer–Emmett–Teller (BET) method of the surface area analyzer (Micromeritics TriStar II 3020). For testing the hydrophobicity, contact angle was determined using OCA drop shape analyzer (Data physics Co., Germany). For the oil adsorbancy test, 0.18 g of poly(TFEMA–DVB) bulk polymer was placed in an oil/water mixture and the polymer was taken out after saturation by adsorption. The total mass of monolith soaked with oil was weighed and the intake capacity of oil, *k* was calculated as follows (eqn (1)):1
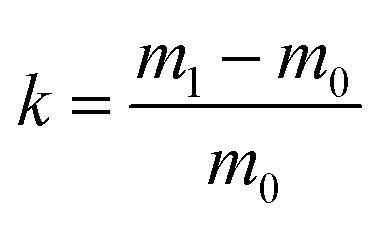
where, *m*_0_ is the mass of monolith before adsorption of oil and *m*_1_ is the mass of monolith after adsorption of oil. Each group of oil adsorption was performed three times and the average was taken. Thermogravimetric analysis (TGA) was performed on a Pyris Diamond TG/DTA (PerkinElmer Co, USA) with a heating rate of 10 °C min^−1^ throughout a temperature range from 50 to 600 °C under nitrogen atmosphere.

## 4. Results and discussion

### 4.1 Formation of stable fluoro-emulsions

**Fig. 2:**
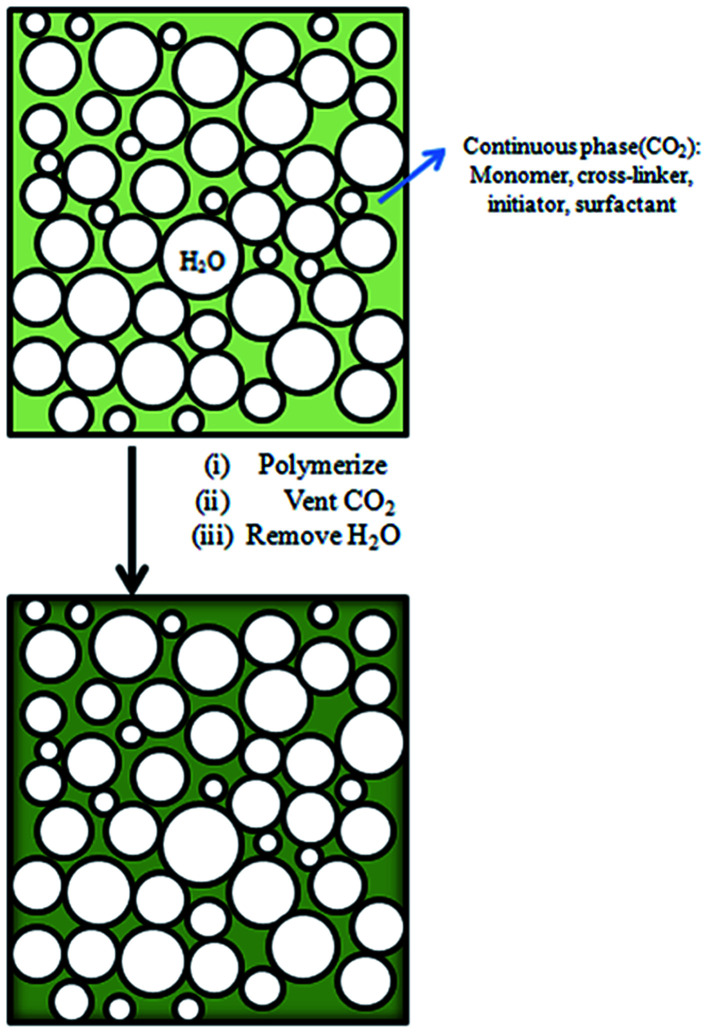
Schematic representing the fabrication of porous materials by W/C emulsion.

In our previous work,^11^ we have successfully prepared porous fluoropolymer materials by W/O HIPE template method. Considering the excellent compatibility between carbon dioxide and fluorinated monomers, sc-CO_2_ was introduced into the emulsion system to prepare fluoropolymer materials. Furthermore, it was investigated for the preparation of W/C emulsion. Supercritical carbon dioxide as a solvent for fluorine-containing monomer, water as an internal phase template, and polyvinyl alcohol (PVA) as a co-surfactant were used in formation of fluorinated emulsions. The main reaction scheme for the preparation of porous fluoropolymer materials using W/C emulsion is shown in Fig. 2. The choice of suitable surfactants is the key to the formation of stable emulsions. Thus, at first the traditional surfactants such as CTAB, SDS, Tween 80, Span 60 and A70 and PVA 2488 as a co-surfactant were selected to prepare stable W/C emulsions. The experiment was also carried out using a self-synthesized amphiphilic block polymer mPEG_45_-*b*-(TFEMA)_80_ as a surfactant to check the performance of surfactant-CO_2_.

**Fig. 3:**
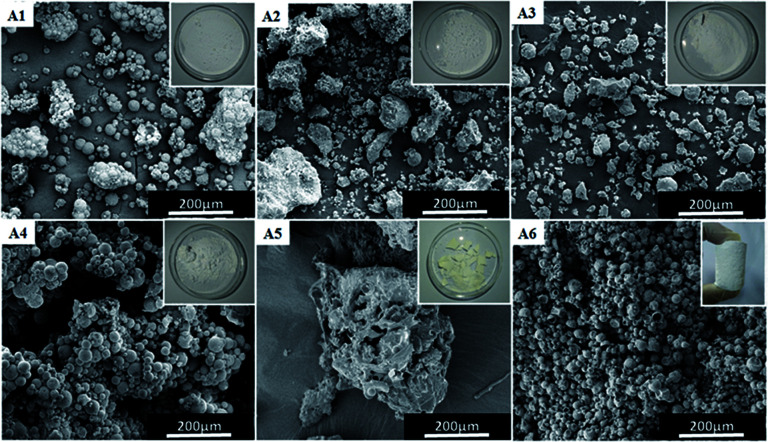
Effects of the type of surfactant on the morphology of poly(TFEMA–DVB) samples (the loading of surfactant is 10 wt% with respect to the monomer phase) as examined by SEM images: (A1) CTAB, (A2) SDS, (A3) Tween 80, (A4) Span 60, (A5) A70, (A6) mPEG_45_-*b*-(TFEMA)_80_.

Fig. 3 shows the photographs and SEM images of fluoropolymer prepared using various conventional surfactants. It can be seen from Fig. 3(A1–A5) that by using these surfactants it becomes difficult to form a complete monolithic columnar structured polymer. By using self-synthesized surfactant mPEG_45_-*b*-(TFEMA)_80_, fluoropolymer formed unitary columnar structure. It can also be proved from SEM images that the conventional surfactant cannot form a stable W/C emulsion. They are different from the SEM image of Fig. 3(A6) and the polymer materials do not form a porous structure as expected. Hong *et al.*^36^ successfully prepared a stable W/O/W multiple emulsion by a one-step method using block polymer PEG-*b*-PS as a surfactant. It is believed that the appearance of a hollow spherical structure is due to the formation of multiple emulsions in the system which may be due to that the hydrophilic segments of the amphiphilic block polymer having a certain degree of CO_2_-philic.

### 4.2 Structure and characterization of (mPEG_45_-*b*-(TFEMA)_*n*_)

The amphiphilic block polymer, mPEG_45_-*b*-(TFEMA)_*n*_ was synthesized by RAFT polymerization using DDMAT as a chain transfer agent, and the characteristics of this polymer are shown in Table 2. The composition of block polymer and structure were confirmed by ^1^H NMR and GPC. GPC was used to characterize the molecular weight (*M*_n_) and its distribution (Fig. S1†). The mean degree of polymerization (*D*_P_) of mPEG_45_-*b*-(TFEMA)_*n*_ block can be calculated by ^1^H NMR spectra (Fig. S2†). Specifically, the mean degree can be calculated by the area ratio of the signal at *δ* 4.25 ppm corresponding to the protons of (CF_3_–C**H**_2_–) and the signal at *δ* 3.30 ppm corresponding to the protons of (–OCH_3_) of mPEG_45_-OH. The characteristic signals of mPEG_45_-DDMAT are 3.80–3.49 (180H, –C**H**_2_O–), 3.46 (2H, –S–C**H**_2_–CH_2_–), 3.37 (3H, C**H**_3_O–), and 2.82 (2H, –S–CH_2_–C**H**_2_–). Coupling efficiency of the prepared block copolymer was very good as can be seen in Fig S2,† it is clear that the peaks of the RAFT coupling agent was present in both first block and di-block copolymer. This indicates that there is even a possibility of adding and couple another block with this copolymer.

**Table 2:** Characteristics of amphiphilic block polymer mPEG_45_-*b*-(TFEMA)_*n*_

*M*_n,th_ a (g mol^−1^)	*M*_n,GPC_ b (g mol^−1^)	*M*_n,NMR_ c (g mol^−1^)	PDI	*D*_P_	Block polymer
12 444	23 947	8196	1.33	35	mPEG_45_-*b*-(TFEMA)_35_
15 804	25 336	9924	1.45	45	mPEG_45_-*b*-(TFEMA)_45_
19 164	28 697	15 800	1.41	80	mPEG_45_-*b*-(TFEMA)_80_
29 244	32 165	19 836	1.42	104	mPEG_45_-*b*-(TFEMA)_104_
35 964	45 315	27 564	1.53	150	mPEG_45_-*b*-(TFEMA)_150_

a The theoretical *M*_n,th_ values were calculated from *M*_n,th_ = *M*_n,macro-CTA_ + *n*M_n,TFEMA_, where *n* represents the degree of polymerization of the polymer, mPEG_45_-*b*-(TFEMA)_*n*_.

b Determined by GPC.

c Determined *via*^1^H NMR spectroscopy analysis of the polymers.

### 4.3 Effect of the length of fluorine-containing segment in the block polymer on the morphology of resultant polymer

As mentioned earlier, it is believed that multiple emulsions are formed due to the hydrophilic segment of the amphiphilic block polymer which has a certain degree of CO_2_-philic. We speculate that the emulsion system also changes with an increase or decrease in the length of fluorine-containing segments (CO_2_-philic segments) in the block polymer, and the morphology of the polymer also changes after polymerization.

**Fig. 4:**
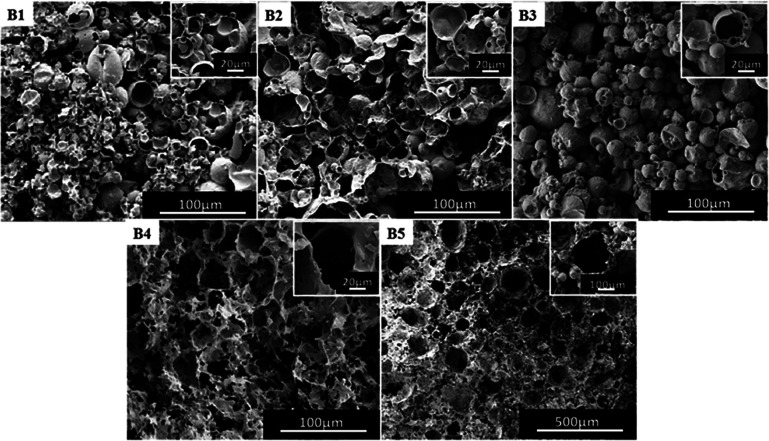
SEM images of poly(TFEMA–DVB) using mPEG_45_-*b*-(TFEMA)_*n*_ surfactant with different fluorine-containing segments as surfactant: (B1) *n* = 35, (B2) *n* = 45, (B3) *n* = 80, (B4) *n* = 104, (B5) *n* = 150.

In order to investigate the effect of the length of fluorine-containing segment on the morphology of the polymer, the block polymers mPEG_45_-*b*-(TFEMA)_*n*_ were synthesized by RAFT polymerization. Five block polymers with different degrees of polymerization (*n* = 35, 45, 80, 104, 150) were selected as the surfactant for emulsion and their corresponding poly(TFEMA–DVB) were prepared by the polymerization. Macroscopically, all of the five groups of experiments produced a complete columnar polymer. However, a large difference in the microstructure of the polymers can be found out through analysing SEM images. The morphology of these polymers can be clearly observed as shown in Fig. 4. In Fig. 4(B1), the presence of small hollow spheres in the large hollow sphere is observed. It can be preliminarily inferred that the emulsion forms the W/C/W/C system. Fig. 4(B1 and (B2) demonstrate same morphology, indicating that the morphology of polymer is stable. Besides, it signifies that the length of the fluorine-containing segment in the block polymer is not enough to change the morphology of the polymer. Further, when the fluorine-containing chain length in the block polymer increases, the morphology of the polymer begins to change (Fig. 4(B3)), where the hollow spherical structure appears. It is expected that the emulsion will be a W/C/W system. The fluorine-containing segment continuously grows and the porous structure begins to appear in the polymer (Fig. 4(B4 and B5)), and W/C emulsion system is formed. We have tested three different structural samples (B1, B3, B4) by FTIR Spectrometer (Fig. S3†) and GPC (Fig. S4†). Three samples are identical in chemical structure. The porous structure (B4, *M*_n_ = 6871, PDI = 1.24) has a lower molecular weight than a small hollow spheres in the large hollow sphere (B1, *M*_n_ = 7122, PDI = 1.28) and hollow spherical (B3, *M*_n_ = 7210, PDI = 1.31). Which indicates that increasing the molecular weight of the block copolymer, the morphology is more likely to form as hollow spheres. Comparatively less molecular weight of block copolymer favors porous polyHIPE morphology as shown in the Fig. 4.

In summary, the growth of the fluorine-containing segment in the block polymer weakens the hydrophilic segment, whereas the CO_2_-philicity of the fluorine-containing segment is enhanced, which results in different types of emulsions and thereby different morphologies of polymers. When the fluorine-containing segment is short (*n* = 35, 45), the hydrophilic segment shows strong CO_2_-philic and also strong hydrophilicity, and thus the emulsion system forms a relatively complex multiple emulsion. When the fluorine-containing segment grows, the CO_2_-philic of the hydrophilic segment is weakened, and the fluorine-containing segment has strong CO_2_-philic, and it gradually forms W/C/W emulsion and then to further forming W/C emulsion.

### 4.4 Effect of the type of cosurfactant PVA on the morphology of the polymer

**Fig. 5:**
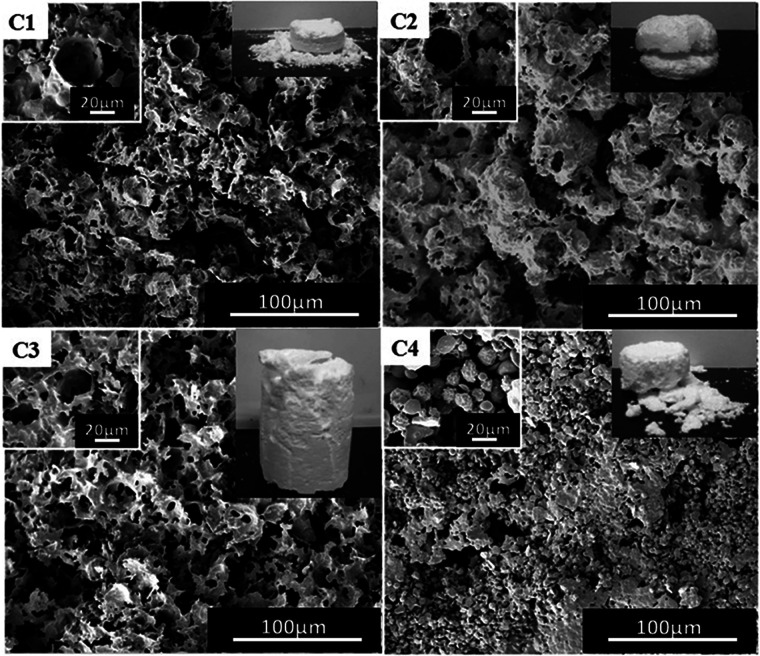
SEM images of the poly(TFEMA–DVB) using the block polymer mPEG_45_-*b*-(TFEMA)_104_ as a surfactant and different co-surfactants; (C1) PVA 1788, (C2) PVA 1799, (C3) PVA 2488, (C4) PVA 2699.

Based on the formation of porous polymer material, the corresponding block polymer mPEG_45_-*b*-(TFEMA)_104_ was selected as the surfactant and the emulsion stability as well as the effects of the morphology of polymer were explored by using different types of PVA (1788, 1799, 2488, 2699). We chose a PVA solution with a mass fraction of 1.5% because the complete columnar polymer is formed at this mass fraction of PVA 2488 compared to other mass fractions. It can be clearly seen from Fig. 5 that the polymer using PVA 2488 as a co-surfactant has a better macroscopic morphology, whereas the polymers show poor morphology by adding PVA of 1788, 1799 and 2699. SEM images demonstrate obtaining pore-like structure using PVA of 1788 and 2488 (Fig. 5(C1) and (C3)), but with PVA of 1799 and 2699 it is difficult to obtain the structure (Fig. 5(C2) and (C4)). This is due to the solubility of OVAc in CO_2_ which is strongly dependent on the molecular weight as the molecular weight of OVAc could affect the hydrophilic–CO_2_-philic balance.^37^ While, at the same mass fraction PVA of 1788 and 2488 contain more OVAc than PVA of 1799 and 2699. With an increase in the molecular weight of PVA, the viscosity of PVA solution at the same concentration is higher and the emulsions are more stable within a specific concentration range of cosurfactant.^21^ At the same concentration, PVA 2488 has a higher viscosity than PVA 1788 and a complete columnar polymer is obtained by using PVA 2488.

### 4.5 Effect of concentration of surfactant mPEG_45_-*b*-(TFEMA)_104_

**Fig. 6:**
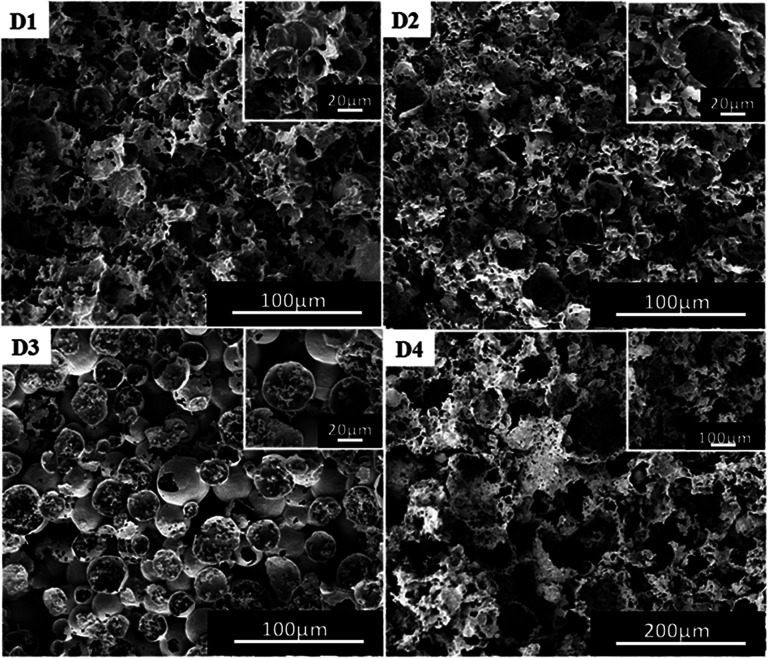
SEM images of poly(TFEMA–DVB) with different contents of surfactant to oil phase; (D1) 3 wt%, (D2) 5 wt%, (D3) 15 wt%, (D4) 20 wt%.

For traditional W/O emulsions, surfactants are one of the important factors to regulate the morphology of polymers.^39^ P(TFEMA–DVB) materials were prepared by adding surfactants of 3 wt%, 5 wt%, 10 wt% and 15 wt%. The content of DVB was 10% by weight of the oil phase, and the selected co-surfactant was PVA 2488. From Fig. 6 and Table S1,† changes in the pore size of polymer could be clearly seen. The pore size of the polymer became irregular when the surfactant used was 3 wt% (Fig. 6(D1)) to 10 wt% (Fig. 4(B4)). Although the pore size of polymer by employing surfactant of 3 wt% is less than applying 5% (Fig. 6(D2)), the obtained polymer was not a complete columnar polymer (Fig. S5(a)†), whereas with other contents a complete columnar polymer can be obtained. It is indicated that surfactant of 3 wt% is insufficient to completely stabilize W/C emulsion, and when the content of the surfactant was 5 wt% and 15 wt%, a stable W/C emulsion was obtained.

Theoretically, the pore size should be further reduced with a further increase in the surfactant, but a different structure was obtained (Fig. 6(D3)). During further investigations, the amount of surfactant was increased to 20 wt% (Fig. 6(D4)), which resulting in the morphology of the polymer to become more irregular as well as difficulty to form a complete columnar polymer (Fig. S3(e)†). The obtained results have shown that an excess amount of surfactant is not conducive to the formation of a stable emulsion. This porous morphological transition can also be explained by the effect of co-surfactant-induced instability of the di-block copolymer *via* covalent crosslinking. When the amount of the diblock copolymer was increased to 20 wt%, the corresponding amount of PVA 2488 became comparatively less to the level that it could not provide enough stability to emulsions. It may be due to the drop in concentration of co-surfactant led to decrease in the ability of the block copolymer to stabilize the emulsion system, which ultimately caused the destabilization of the resulting HIPE.^38^ This created irregular final polyHIPE morphology, at more than 15 wt% of block copolymer.

### 4.6 Effect of the amount of crosslinker

**Fig. 7:**
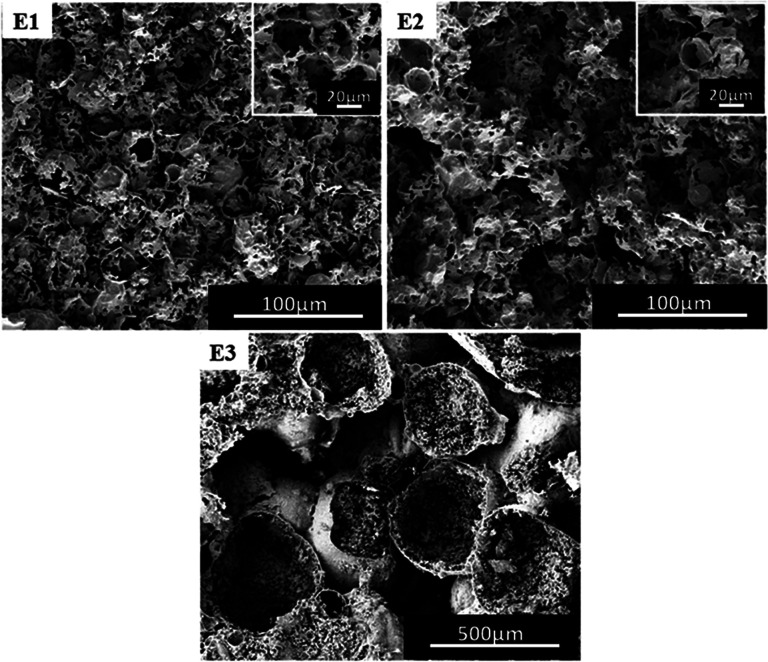
SEM images of poly(TFEMA–DVB) with different contents of DVB (relative to the oil); (E1) 20 wt%, (E2) 30 wt%, (E3) 50 wt%.

In general, the content of cross-linking monomers can also have an effect on the morphology of the polymer.^40,41^ In the experiments, the content of DVB relative to the oil was selected as 10 wt% (Fig. 4(B4)) and 20 wt%, 30 wt% as well as 50 wt% (Fig. 7). When the content of DVB was between 10 wt% and 30 wt%, the polymer also had porous characteristics, but only with heterogeneity (Fig. 7(E1)). Further, the presence of an irregular morphology could be noted (Fig. 7(E2)*vs.*Fig. 7(E1)), along with the absence of porous structure (Fig. 7(E3)). It has been found that with an increase in the concentration of DVB cross-linker, W/C emulsion became non-uniform, as the DVB cross-linker does not have good compatibility with TFEMA and CO_2_. With an increase in DVB, the solubility of oil phase in CO_2_ decreases, affecting the uniform stability of W/C emulsion. Since all the samples B4, D1, D2 and E1 have porous morphologies, we performed a BET test (Table S1†) to compare the difference in the pores to determine which formulation is superior. From Table S1,† (B4) has a larger specific surface area and a smaller pore size.

### 4.7 Effect of water/CO_2_ ratio on the porous structure

**Fig. 8:**
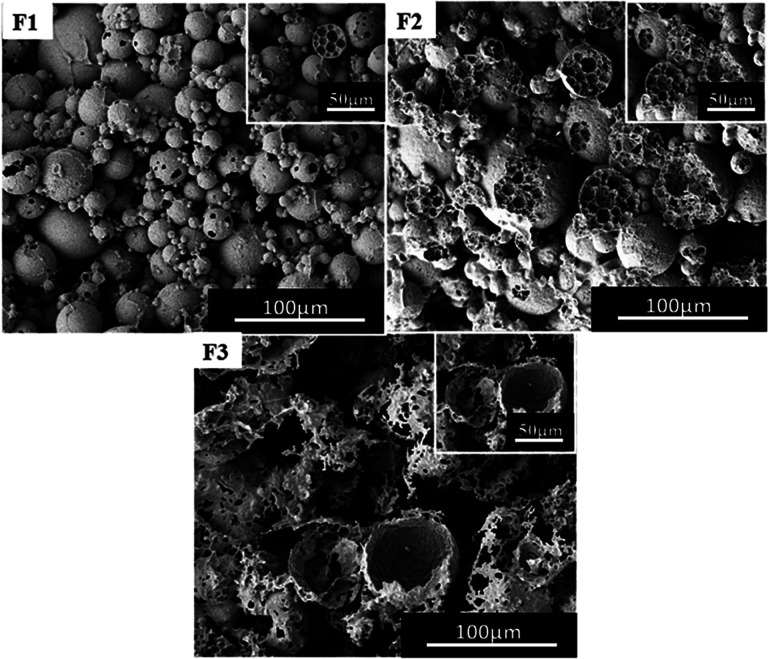
SEM images of poly(TFEMA–DVB) with different aqueous phase fractions; the amount of CO_2_ is constant at 10 g; (F1) 15 ml, (F2) 25 ml, and (F3) 30 ml.

In the above experiments, the volume of water used was 38 ml (the volume of autoclave was 50 ml), and the amount of CO_2_ was 10 g. For ordinary emulsions, water/oil ratio has a critical impact on the morphology of the polymer. At first a reduced volume of water was used, for example 15 ml, 25 ml and 30 ml water (including PVA 2448) and the obtained experimental results are shown in Fig. 8(F1–F3). From the SEM images, it can be observed that the morphology of the polymer changes with the volume of water, *i.e.* the type of emulsion changes from W/C to W/C/W. This is similar to the observations of Torino^42^ and Salager^43^ where they obtained different types of emulsion by changing the ratio of water to CO_2_.

However, when the water content reduced from 4 to 2% by using the surfactant Tween 80, the form of emulsion changed from W/C to C/W to C/W/C.^41^ From Fig. 4(B4) to Fig. 8(F1–F3) a change in the emulsion system from W/C to W/C/W could be noted. Considering that the block polymer mPEG_45_-*b*-(TFEMA)_104_ is stable better in water/CO_2_ than in Tween 80, there will be differences in the obtained experimental results. The experiments were repeated to adjust the amount of water in order to get the same results.

### 4.8 Effect of macro-CTA/initiator ratio on the porous structure

We also explored the preparation of block polymers at different ratios of macro-chain transfer agent and initiators (macro-CTA/AIBN = 1, macro-CTA/AIBN = 2, macro-CTA/AIBN = 4, macro-CTA/AIBN = 5). In the preparation of the block polymer, the formulation is consistent with the preparation of mPEG_45_-*b*-(TFEMA)_104_.

As the concentration of initiator increases (macro-CTA/AIBN = 1, macro-CTA/AIBN = 2), the amount of free radicals increases, the rate of polymerization increases, and the probability of chain termination increases.^44^ The PDI of the block polymer is broadened and the degree of polymerization is decreased. This phenomenon is clearly observed in GPC (Fig. S7(a)†).

Macro-CTA transfers the free radical to the structure (–S(C=S)S–R) to form an active species and a dormant species.^45^ As the amount of initiator decreases (macro-CTA/AIBN = 4, macro-CTA/AIBN = 5), the amount of free radicals is further reduced, making the initiation process slow. We increased the time (48 h) to synthesize block polymers, the molecular weight of the block polymer was similar to the molecular weight of the macro-CTA by Fig. S7(b),† also the yield of block polymer was very low (<10%), this indicates that the block polymer mPEG_45_-*b*-(TFEMA)_*n*_ has little or no fluorine-containing end, hence it can be concluded that the formation of block copolymers at low initiator contents is not successful.

**Fig. 9:**
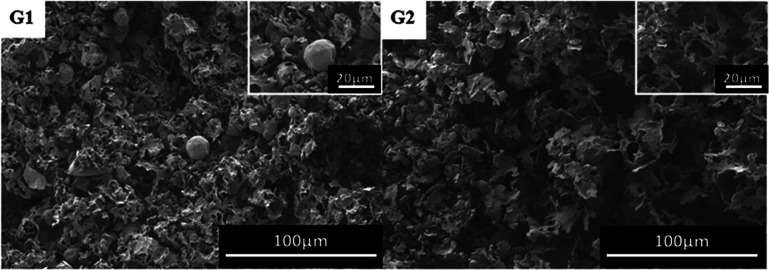
SEM images of poly(TFEMA–DVB) with different ratios of macro-CTA and initiator; (G1) macro-CTA/AIBN = 1, (G2) macro-CTA/AIBN = 2.

We have prepared a fluorine-containing material by using a block polymer prepared under macro-CTA/AIBN = 1 and macro-CTA/AIBN = 2 as a surfactant. From Fig. 9, the polymer has a semi-porous structure. This is due to the broad molecular weight distribution of diblock copolymers.

### 4.9 Hydrophobicity and adsorption ability of the oil of P(TFEMA–DVB) foam

**Fig. 10:**
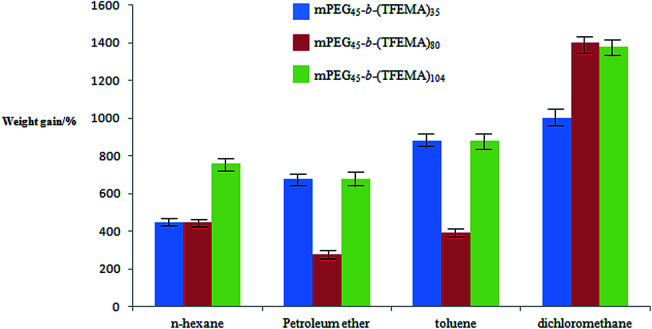
The adsorption capacity of three structural polymers poly(TFEMA–DVB) prepared using various organic solvents.

In our previous studies we have demonstrated that fluoropolymer materials have excellent hydrophobicity and lipophilicity. Because of the presence of –CF_3_– group in TFEMA which has a low surface energy which is strictly required for the material to have hydrophobicity or lipophilicity, and also used in preparation of di-block copolymers.^46,47^ For the polymers with three different morphology prepared by three surfactants (Fig. 4(B1), (B3) and (B4)), four oil phases (*n*-hexane, petroleum ether, toluene and dichloromethane) were selected for testing the adsorption of oil. It can be seen from Fig. 10 that the porous structure exhibits better lipophilicity in the four oil phases compared to other two structures. Whereas, the hollow spherical structure exhibits better lipophilicity in dichloromethane, and the lipophilicity in other three oil phases is weaker compared to other two structures.

**Fig. 11:**
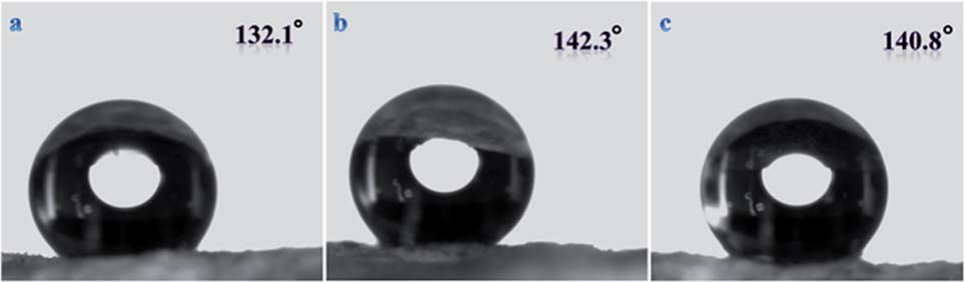
Water contact angle of polymer materials of three structures prepared using three different surfactants; (a) mPEG_45_-*b*-(TFEMA)_35_, (b) mPEG_45_-*b*-(TFEMA)_80_, (c) mPEG_45_-*b*-(TFEMA)_104_.

The test for the hydrophilicity of three structures was carried out using a water contact angle instrument. As shown in Fig. 11, the porous and the hollow spherical structures have similar hydrophilicity, while hydrophobicity is poor for the material with small hollow spherical structure. By comparing Fig. 10 and 11, the experiment can give the result that different structures have a great influence on the lipophilicity and hydrophobicity of the material. In general, the porous structure has a better performance in lipophilicity and hydrophobicity.

### 4.10 Chemical resistance and thermal stability of fluoromonolith

Three samples with different morphologies (B1, B3, B4) were dipped into acidic and basic aqueous solutions to test chemical resistance of fluoropolymers. Clips were used to ensure good sinking of samples into solution.

**Fig. 12:**
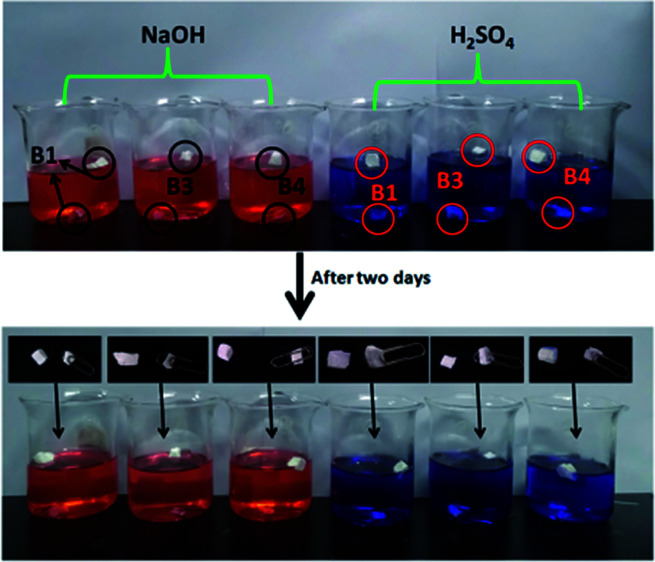
Photographs of immersion of three samples with different morphologies in alkali (1 mol l^−1^ NaOH solution dyed with methyl orange) and acids (1 mol l^−1^ H_2_SO_4_ dyed with methylene blue).

**Fig. 13:**
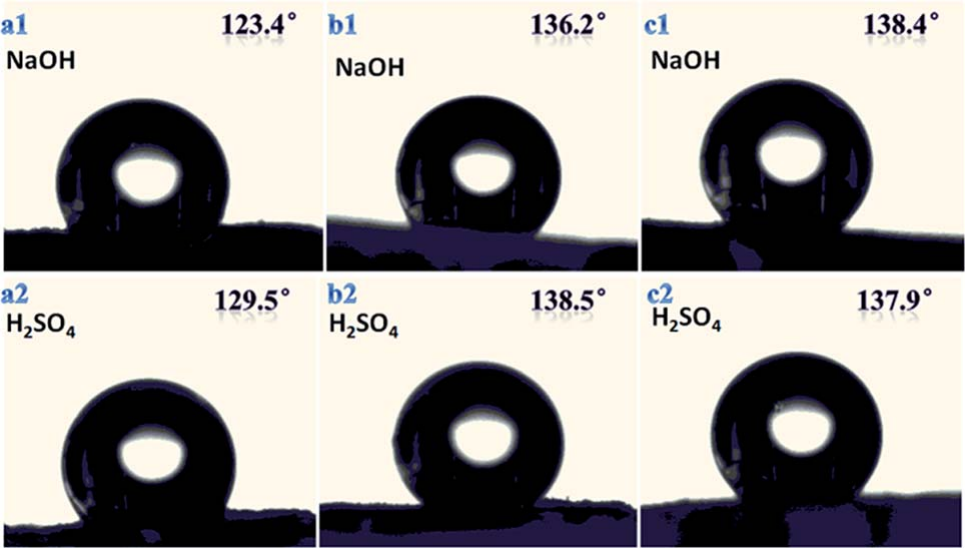
Water contact angle for three samples with different morphologies in chemical environment for 2 days; (a1) and (a2) sample B1, (b1) and (b2) sample B3, (c1) and (c2) sample B4.

We found that there was no obvious swelling or no fragmentation to the surface of three samples after immersion in acidic or basic solution for two days as shown in Fig. 12. From Fig. 11 and 13 water contact angles were not significantly dropped. This indicates that the material has excellent corrosion resistance and can be applied under harsh conditions.

**Fig. 14:**
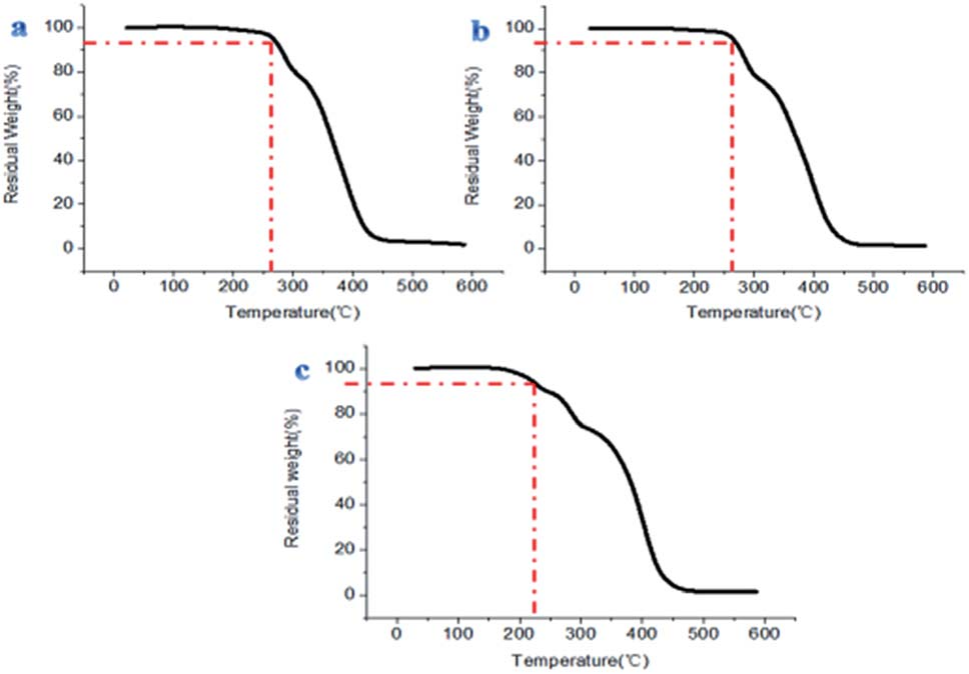
TGA curves of (a) sample B1, (b) sample B3 and (c) sample B4.

The prepared materials having small hollow spheres in the large hollow sphere or hollow spherical structure showed remarkable stability performance at about 260 °C, and the materials with porous structures also demonstrated good heat resistance below 220 °C (Fig. 14). This shows that different fluoropolymers prepared in this report have superb ability to be used under high temperature conditions.

## 5. Conclusions

In this study, amphiphilic block polymer mPEG_45_-*b*-(TFEMA)_*n*_ was synthesized by RAFT polymerization, and the block polymer was used as a surfactant for the generation of emulsion, and sc-CO_2_ was used as a solvent for the fluorine-containing monomer. W/C emulsion was prepared to further generate a porous fluoropolymer. During the experiment, when different lengths of fluorine-containing segment (CO_2_-philic segments) were used as surfactants, three different structures of polymeric materials were prepared: small hollow spheres in the large hollow sphere, hollow spherical structure, and porous structure. It is speculated that different emulsion types are formed. Finally, the control of different morphologies of the polymer can be achieved by controlling the size of “*n*” in the amphiphilic block polymer mPEG_45_-*b*-(TFEMA)_*n*_.

At the same time, based on the preparation of C/W emulsion, the stability of the emulsion and the shape of the polymer can be controlled from the selection of different co-surfactants,^47^ adjusting the concentration of surfactant, amount of crosslinker and the ratio of water to CO_2_. However, unexpected problems were noted in the experiments, when the concentration of surfactant was higher than 15%, whereby the polymer exhibited a large transformation in the morphology and the stability of emulsion became poor. The morphology of the polymer was different with an adjustment in water/CO_2_ ratio.

## Conflicts of interest

Author declares no conflict of interest for this research paper.

## Supplementary Material

RA-009-C9RA00777F-s001

## References

[cit1] Luo W., Xu R., Liu Y., Hussain I., Lu Q., Tan B. (2015). RSC Adv..

[cit2] Moglia R. S., Holm J. L., Sears N. A., Wilson C. J., Harrison D. M., Cosgriff-Hernandez E. (2011). Biomacromolecules.

[cit3] Errahali M., Gatti G., Tei L., Paul G., Rolla G. A., Canti L., Fraccarollo A., Cossi M., Comotti A., Sozzani P., Marchese L. (2014). J. Phys. Chem. C.

[cit4] He H., Li W., Lamson M., Zhong M., Konkolewicz D., Hui C. M., Yaccato K., Rappold T., Sugar G., David N. E., Damodaran K., Natesakhawat S., Nulwala H., Matyjaszewski K. (2014). Polymer.

[cit5] Buyukcakir O., Je S. H., Choi D. S., Talapaneni S. N., Seo Y., Jung Y., Polychronopoulou K., Coskun A. (2016). Chem. Commun..

[cit6] Debecker D. P., Boissiere C., Laurent G., Huet S., Eliaers P., Sanchez C., Backov R. (2015). Chem. Commun..

[cit7] Sun Q., Dai Z., Meng X., Xiao F.-S. (2015). Chem. Soc. Rev..

[cit8] Pulko I., Wall J., Krajnc P., Cameron N. R. (2010). Chem.–Eur. J..

[cit9] Zhang Y., Pan J., Chen Y., Shi W., Yan Y., Yu L. (2016). Chem. Eng. J..

[cit10] Walcarius A., Mercier L. (2010). J. Mater. Chem..

[cit11] Wan X., Azhar U., Wang Y., Chen J., Xu A., Zhang S., Geng B. (2018). RSC Adv..

[cit12] De Simone J. M. (2002). Science.

[cit13] Nalawade S. P., Picchioni F., Janssen L. P. B. M. (2006). Prog. Polym. Sci..

[cit14] Butler R., Hopkinson I., Cooper A. I. (2003). J. Am. Chem. Soc..

[cit15] Dhanuka V. V., Dickson J. L., Ryoo W., Johnston K. P. (2006). J. Colloid Interface Sci..

[cit16] Stone M. T., da Rocha S. R. P., Rossky P. J., Johnston K. P. (2003). J. Phys. Chem. B.

[cit17] Dalvi V. H., Srinivasan V., Rossky P. J. (2010). J. Phys. Chem. C.

[cit18] Boyere C., Leonard A. F., Grignard B. (2012). Chem. Commun..

[cit19] Partap S., Rehman I., Jones J. R., Darr J. A. (2006). Adv. Mater..

[cit20] Butler R., Davies C. M., Cooper A. I. (2001). Adv. Mater..

[cit21] Luo W., Xu R., Liu Y., Hussain I., Lu Q., Tan B. (2015). RSC Adv..

[cit22] Luo W., Zhang S., Li P., Xu R., Zhang Y., Liang L., Wood C. D., Lu Q., Tan B. (2015). Polymer.

[cit23] Harrison K., Goveas J., Johnston K. P. (1994). Langmuir.

[cit24] Su B., Xing H., Ren Q. L. (2008). J. Chem. Eng. Data.

[cit25] Campbell M. L., Apodaca D. L., Yates M. Z. (2001). Langmuir.

[cit26] Hanrahan J. P., Zeigler K. K. J., Galvin J. P., Homes J. D. (2004). Langmuir.

[cit27] Lim K. T., Hwang H. S., Ryoo W. (2004). Langmuir.

[cit28] Braunecker W. A., Matyjaszewski K. (2007). Prog. Polym. Sci..

[cit29] Discekici E. H., Anastasaki A., Kaminker R., Willenbacher J., Truong N. P., Fleischmann C., Oschmann B., Lunn D. J., de Alaniz J. R., Davis T. P., Bates C. M., Hawker C. J. (2017). J. Am. Chem. Soc..

[cit30] Anastasaki A., Oschmann B., Willenbacher J., Melker A., Van Son M. H. C., Truong N. P., Schulze M. W., Discekici E. H., McGrath A. J., Davis T. P., Bates C. M., Hawker C. J. (2017). Angew. Chem., Int. Ed..

[cit31] Ma J., Zhang L., Geng B., Azhar U., Xu A., Zhang S. (2017). Molecules.

[cit32] Truong N. P., Whittaker M. R., Anastasaki A., Haddleton D. M., Quinn J. F., Davis T. P. (2016). Polym. Chem..

[cit33] Truong N. P., Quinn J. F., Anastasaki A., Rolland M., Vu M. N., Haddleton D. M., Whittaker M. R., Davis T. P. (2017). Polym. Chem..

[cit34] Azhar U., Huyan C., Wan X., Xu A., Li H., Geng B., Zhang S. (2017). Mater. Des..

[cit35] Lai J. T., Filla D., Shea R. (2002). Macromolecules.

[cit36] Hong L., Sun G., Cai J., Ngai T. (2012). Langmuir.

[cit37] Chen K., Grant N., Liang L., Zhang H., Tan B. (2010). Macromolecules.

[cit38] Truong N. P., Zhang C., Nguyen T. A. H., Anastasaki A., Schulze M. W., Quinn J. F., Whittaker A. K., Hawker C. J., Whittaker M. R., Davis T. P. (2018). ACS Macro Lett..

[cit39] Wong L. L. C., Baiz Villafranca P. M., Menner A., Bismarck A. (2013). Langmuir.

[cit40] Sevšek U., Brus J., Jeřabek K., Krajnc P. (2014). Polymer.

[cit41] Nalawade A. C., Ghorpade R. V., Shadbar S., Qureshi M. S., Chavan N. N., Khan A. A., Ponrathnam S. (2016). J. Mater. Chem. B.

[cit42] Torino E., Reverchon E., Johnston K. P. (2010). J. Colloid Interface Sci..

[cit43] Salager J. L., Forgiarini A., Marquez L., Pena A., Pizzino A., Rodriguez M. P., Rondo-Gonzalez M. (2004). Adv. Colloid Interface Sci..

[cit44] Moad G., Mayadunne R. T. A., Rizzardo E., Skidmore M., Thang S. H. (2002). ACS Symp. Ser..

[cit45] Goto A., Sato K., Tsujii Y., Fukuda T., Moad G., Rizzardo E., Thang S. H. (2001). Macromolecules.

[cit46] Azhar U., Yaqub R., Li H., Abbas G., Wang Y., Chen J., Zong C., Xu A., Yabin Z., Zhang S., Geng B. (2019). Arabian J. Chem..

[cit47] Azhar U., Zong C., Wan X., Xu A., Yabin Z., Liu J., Zhang S., Geng B. (2018). Chem.–Eur. J..

