# Supplementary material for: Preparation of fluoropolymer materials with different porous morphologies by an emulsion template method using supercritical carbon dioxide as a medium
Source: RSC Adv. 2019 Apr 11;9(20):11331–40. doi: 10.1039/c9ra00777f (PMC9063414; doi:10.1039/c9ra00777f)
Supplement: RA-009-C9RA00777F-s001 [file RA-009-C9RA00777F-s001.pdf]

Electronic Supplementary Material (ESI) for RSC Advances.
This journal is © The Royal Society of Chemistry 2019

Supplementary Information

Preparation of fluoropolymer materials with different porous morphologies by emulsion template method using supercritical carbon dioxide as a medium

Jian Chen, [†a] Umair Azhar, [†a] Yongkang Wang, [b] Jihong Liang, [b] and Bing Geng *[b]

a. Shandong Provincial Key Laboratory of Fluorine Chemistry and Chemical Materials.

b. Institute of Fluorescent Probes for Biological Imaging, University of Jinan, Shandong, China

School of Chemistry and Chemical Engineering, University of Jinan, Jinan 250022, China.

E-mail: chm_gengb@ujn.edu.cn

† *Jian Chen and Umair Azhar contributed equally to this work*

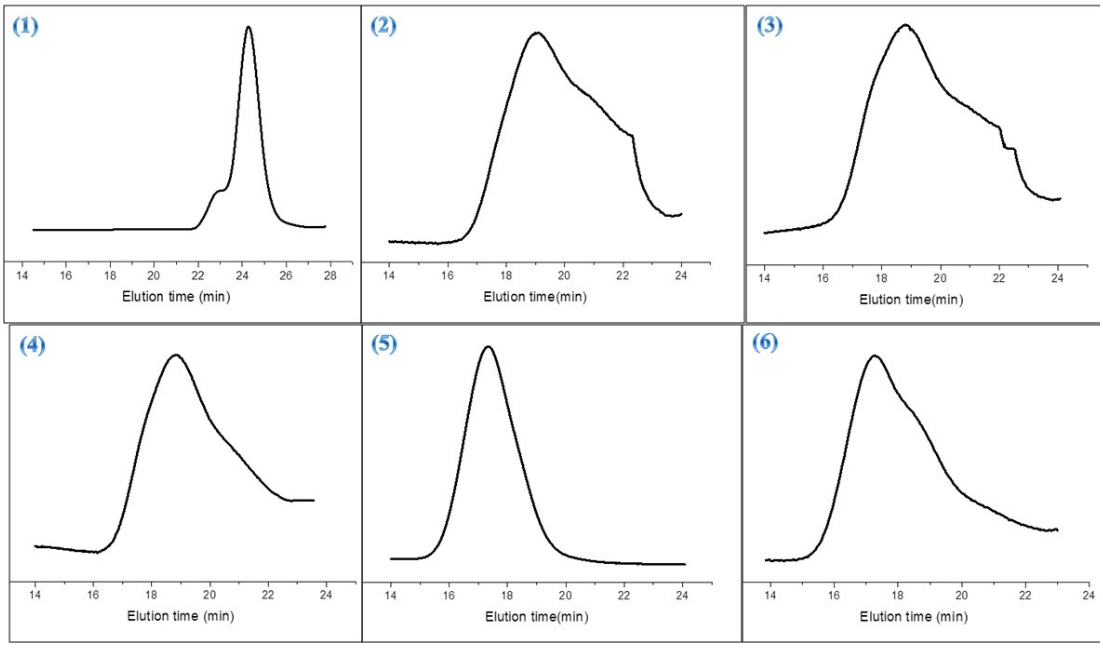

Fig. S1 GPC curves of: (1) $mPEG_{45}$-DDMAT, (2) $mPEG_{45}$-b-$(TFEMA)_{35}$, (3) $mPEG_{45}$-b-$(TFEMA)_{45}$, (4) $mPEG_{45}$-b-$(TFEMA)_{80}$, (5) $mPEG_{45}$-b-$(TFEMA)_{104}$, (6) $mPEG_{45}$-b-$(TFEMA)_{150}$.

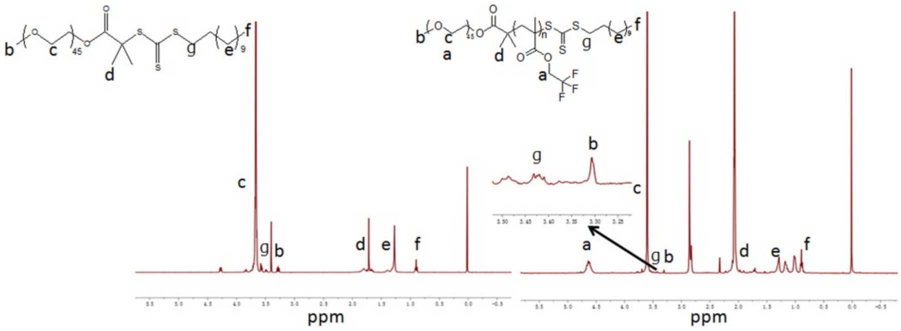

Fig. S2 ^{1}H NMR curves of: (1) $mPEG_{45}$-DDMAT, and (2) $mPEG_{45}$-b-$(TFEMA)_n$; Magnified peaks indicates the presence of RAFT agent even after formation of di-block copolymer

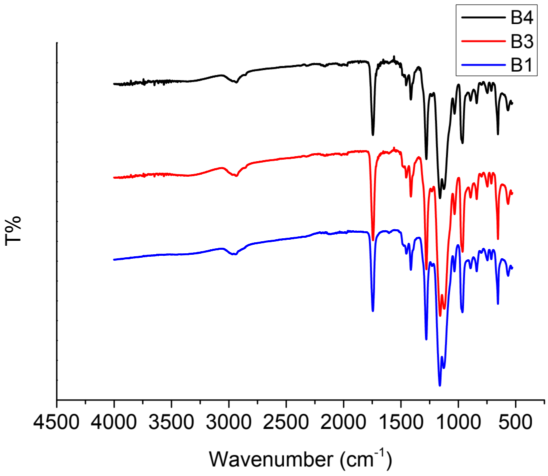

Fig. S3 FTIR spectrum of different morphological sample(B4, B1, B3).

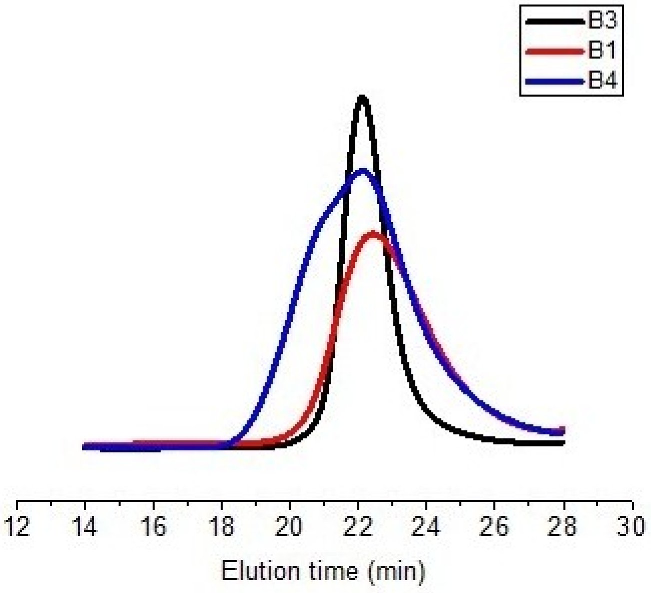

Fig. S4 GPC curves of different morphological sample(B4, B1, B3).

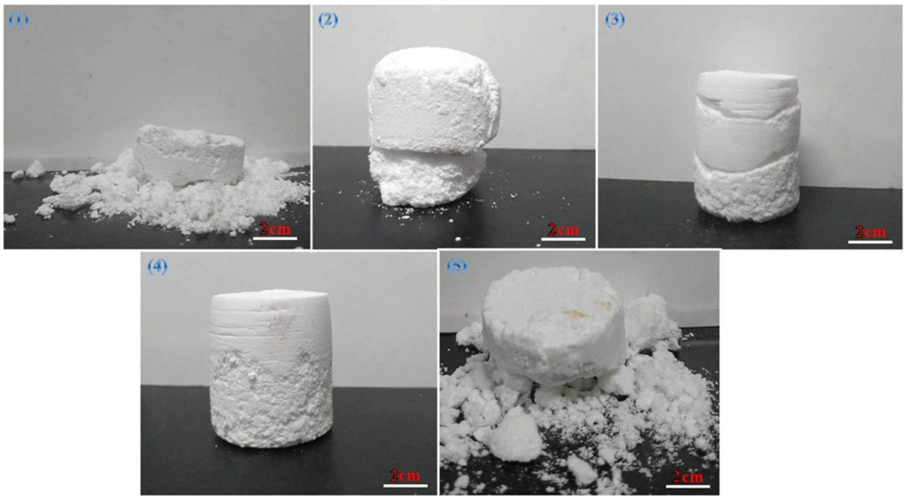

Fig. S5 Photographs of a polymer poly(TFEMA-DVB) formed by using different amounts of surfactant: (1) 3wt%, (2) 5wt%, (3) 10wt%, (4) 15wt%, (5) 20wt%.

Table S1 Foam average void diameter (D_v) of porous polymer.

Sample	TFEMA/DVB mass ratio	$mPEG_{45}$-$(TFEMA)_{104}$ **(wt%)**	CO_2(g)	2488 PVA aqueous(1.5% mass fraction) (ml)	D_v [μm]	BET surface area (m^2g^{-1})
B4	0.9	10	10	38	15.82	28.23
D1	0.9	3	10	38	14.44	15.22
D2	0.9	5	10	38	21.24	27.89
E1	0.8	10	10	38	19.99	12.91

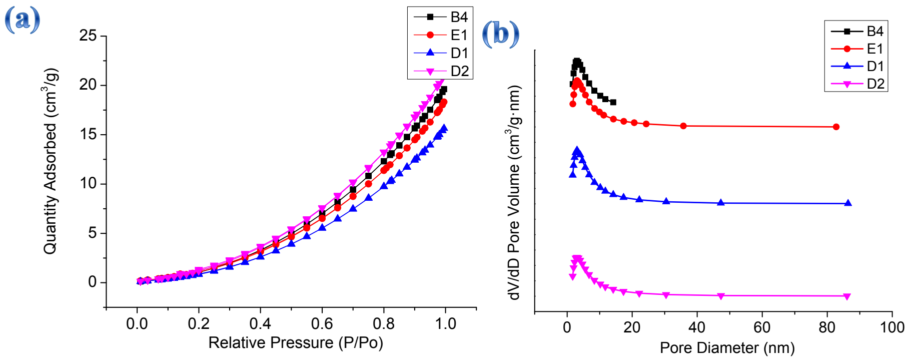

Fig. S6 BET nitrogen adsorption–desorption isotherms (a)and pore size distributions (b) of different morphological sample(B4, E1, D1, D2).

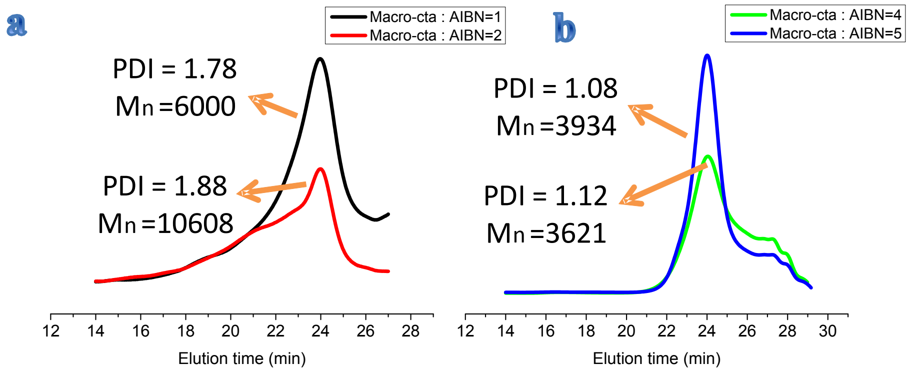

Fig. S7 GPC curves of the block polymers $mPEG_{45}$-*b*-$(TFEMA)_n$ prepared at different ratios of macro-CTA and initiator.
